# The point-spread function of fiber-coupled area detectors

**DOI:** 10.1107/S0909049512035571

**Published:** 2012-09-05

**Authors:** James M. Holton, Chris Nielsen, Kenneth A. Frankel

**Affiliations:** a Department of Biochemistry and Biophysics, University of California, San Francisco, CA 94158-2330, USA; b Lawrence Berkeley National Laboratory, Berkeley, CA 94720, USA; c Area Detector Systems Corporation, Poway, CA 92064, USA

**Keywords:** protein crystallography X-ray detector, CCD, phosphor, fiber optic

## Abstract

The point-spread function (PSF) of a fiber-optic taper-coupled CCD area detector was measured over five decades of intensity using a 20 µm X-ray beam and ∼2000-fold averaging. The ‘tails’ of the PSF clearly revealed that it is neither Gaussian nor Lorentzian, but instead resembles the solid angle subtended by a pixel at a point source of light held a small distance (∼27 µm) above the pixel plane. This converges to an inverse cube law far from the beam impact point. Further analysis revealed that the tails are dominated by the fiber-optic taper, with negligible contribution from the phosphor, suggesting that the PSF of all fiber-coupled CCD-type detectors is best described as a Moffat function.

## Abstract 2

The point-spread function (PSF) of a powdered-phosphor fiber-optic taper-coupled CCD area detector was measured, and an analytic expression for its functional form proposed. The ‘tails’ of this PSF were shown to be due almost entirely to the fiber-optic taper, with no contribution from the phosphor itself.

## 1. Introduction

The signal-to-noise ratio of weak diffraction spots is limited by the background-scattered photons that fall into the spot-integration area (Arndt & Wonacott, 1977 ▶; Holton & Frankel, 2010 ▶), and the size of this area is, in turn, limited by the point-spread function (PSF) of the detector. By definition, the PSF is the spatial distribution of the recorded signal when the detector is illuminated by an infinitesimally thin beam of light. In practice the PSF is usually measured with the smallest beam available and the shape of the incident beam is deconvoluted from the measured intensity distribution (see §2.3).

It is often reported in the scientific literature and in detector product literature that the PSF of detectors is Gaussian (Madden *et al.*, 2006 ▶), as would be expected from the central limit theorem (de Moivre, 1738 ▶; Hald, 1998 ▶), but if the PSF does not arise from a large number of independent random processes it may have a different shape. Some detectors have been found to produce Lorentzian-shaped peaks (Bourgeois *et al.*, 1994 ▶), but the functional form of the PSF found here is different from both of these: an inverse cube law. One report (Schreurs *et al.*, 2010 ▶) presented a functional form identical to that found here, but did not explain how it was obtained.

### 1.1. Detector construction

The detector studied here was an Area Detector Systems (Poway, CA, USA) model Quantum 315r (serial number 926). These detectors are a 3 × 3 tiled array of nine sub-detectors, each of which consists of a thermoelectrically cooled Amtel THX 7899 CCD chip hard epoxy bonded to the narrow end of a 3.7:1 glass fiber-optic taper (Incom, Charlton, MA, USA). The X-ray-sensitive phosphor is a thin layer (∼20–40 µm) of powdered Gd_2_O_2_S:Tb (<5 µm particles) sandwiched between the wide end of the taper and a thin aluminized black plastic front window. The CCDs and tapers are held in a vacuum, for which the front window is the seal. This vacuum seal holds the phosphor in place. X-ray photons pass though the front window and, upon absorption in the phosphor, ∼10% of the photon energy is converted into hundreds of visible-light photons, which are emitted in random directions. The visible photons scatter about among the phosphor particles (possibly reflecting off the inner surface of the front window) until ultimately being either absorbed or accepted into one of the optical fibers of the taper (reviewed by Gruner *et al.*, 2002 ▶). Detailed simulations of this process have been conducted by Liaparinos *et al.* (2006 ▶).

**Figure 1:**
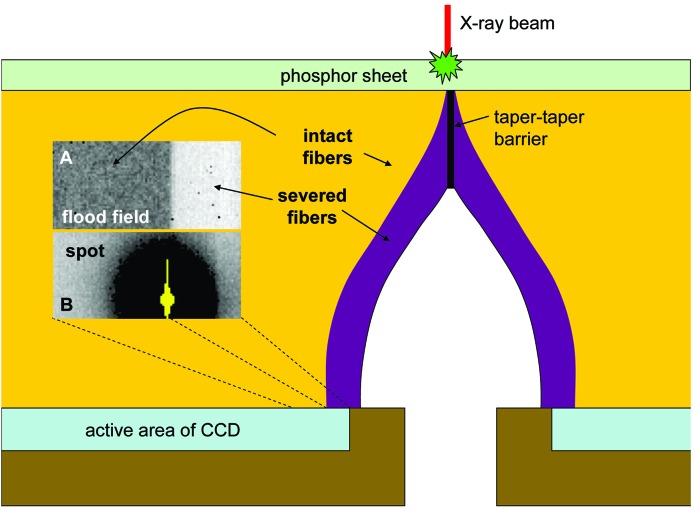
Schematic diagram of the detector construction. Yellow areas indicate ‘intact’ fibers which connect the CCD to the phosphor, and the purple region represents ‘severed’ fibers that contact the CCD but never reach the phosphor. Inset images are taken from the ‘raw’ CCD readout and demonstrate that light created by X-rays diffuses from the intact fibers into the severed fibers and onto the unused CCD pixels.

The fiber-optic tapers consist of millions of thin (10 µm-diameter) optical fibers formed into a round billet that is then heated and pulled into an hourglass shape. This hourglass is then cut into two tapers, which are round, so packing them into a square array requires that the large end be cut down to a square. The narrow end of the taper will eventually be bonded to a CCD chip so must also be square, but care must be taken at this end not to over-cut and damage ‘intact’ fibers that actually directly connect to the phosphor end. A ‘buffer zone’ of about 5% of the total area is therefore left around the square ‘intact region’ on the narrow end. The fibers in this buffer zone are called ‘severed’ here because they terminate on one of the four lateral walls of the squared-off taper, nowhere near the phosphor. The intact region is made to be slightly smaller than the active area of the CCD to allow for small alignment errors when the CCD is bonded to the taper (see Fig. 1 ▶), so the CCD pixels around the edges of the chip are actually bonded to severed fibers. In this way, every point on the phosphor face of the taper is directly connected to the CCD, but not every point on the CCD is connected to phosphor. We took advantage of this quirk in the detector construction to separate the relative contribution of the fiber-optic taper and the phosphor to the total PSF.

## 2. Methods

### 2.1. Experimental set-up

A 50 µm-thick disk of tantalum (Ta) with a 15 µm circular laser-drilled hole in the center was purchased from National Aperture (Salem, NH, USA), and placed at the sample position in the protein crystallography beamline 8.3.1 at the Advanced Light Source [instrument described by MacDowell *et al.* (2004 ▶)]. The photon energy was set to just above the Ta *L*_2_-edge at 11141 eV to maximize the stopping-power contrast of the pinhole, and the convergence angles of the beam were reduced to 50 µrad × 50 µrad by adjusting a set of slits 10 m up-beam from the X-ray focus (pinhole position). The beam stop was removed and the Quantum 315r detector positioned 85 mm from the pinhole. After inserting absorbers, it was found that a 0.1 s exposure yielded a ‘direct-beam’ spot with peak pixel intensity of approximately 20000 pixel levels or ‘analog to digital units’ (ADUs) on an unbinned, dezingered and spatially corrected image. A total of 1883 such direct-beam shots were collected, and each was followed by an equivalent ‘explicit dark’ exposure where the shutter was not opened. These explicit dark images were necessary because if the same dark image were subtracted from all the ‘light’ images then the noise in the common dark image would dominate the analysis below.

The distance between the pinhole and detector minimized the contributions of fluorescence and scattering from the pinhole to a negligible level (see §3.2). Each image was collected with the detector driven to a slightly different position relative to the X-ray beam: ranging at random over an area approximately one pixel wide and six pixels high (the pixel size was 51.3 µm). These movements were executed to sample more than just a single part of a single pixel on the detector surface, but at the same time involve only the central region of one fiber-optic taper. A 200 × 200 pixel region-of-interest (ROI), centered on the spot, was extracted from each image and the corresponding explicit-dark image pixels subtracted to form a ‘net’ image with no read-out noise events in common with any other. The ROI was centered 2 mm × 2 mm down and left of the center of the middle detector module, so all the pixels in this experiment were digitized by the same read-out channel.

**Figure 2:**
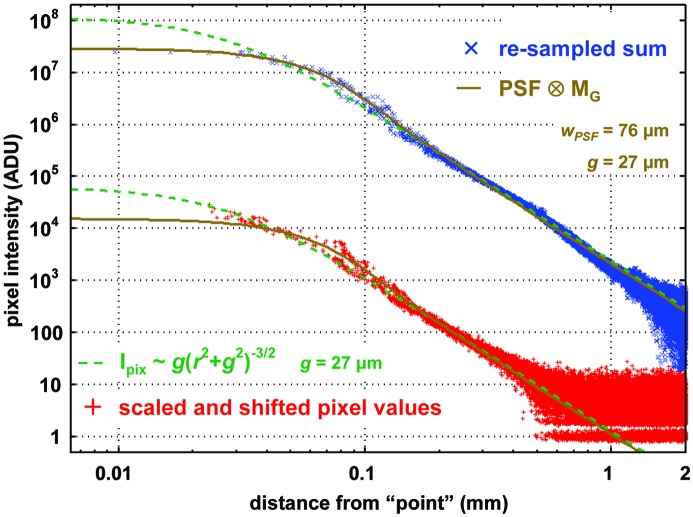
Scaled pixel intensities obtained by subtracting ‘light’ and ‘dark’ detector images are plotted as red points. The *x*-axis (*r*) denotes the distance of the center of each pixel from the fitted center of intensity for the given pixel field. Blue points were obtained by re-binning and summing all intensity data, revealing that the inverse cube law continues out to at least 2 mm away from the beam impact point. The brown solid lines are the best fit to the PSF convoluted with a Gaussian and the green dashed lines are the ‘deconvoluted’ solid-angle component: function *P*.

Each net image was then fitted to a two-dimensional Gaussian function to roughly establish the fractional pixel coordinate of the center of the incident beam and also to obtain a rough scale factor from the height of each fitted function. Using these fitted parameters the midpoint of each pixel could then be assigned a linear distance from the ‘beam center’ and each spot put on a common scale with the others. These shifted and scaled data were then plotted as the red points in Fig. 2 ▶. Scale factors ranged from 0.75 to 1.35 and were due largely to variations in storage-ring current. Note that the grouping of pixel values at unity is a discretization artifact arising because the difference between any two integer-valued pixels must also be an integer.

To improve the signal-to-noise ratio in the low end, each pixel was treated as a square area of constant intensity and the intensity re-distributed onto a new common pixel grid using triangle binning with the program *FIT2D* (Hammersley, 1997 ▶). The resulting ‘sum pixel’ intensities are plotted as the blue points in Fig. 2 ▶. This peak-fitting and re-binning procedure was repeated using the model for the actual PSF derived below to extract the center and scale of each observed spot, but the resulting changes to the points plotted in Fig. 2 ▶ were unremarkable.

### 2.2. Assessment of fiber-optic taper contribution

Separating the contributions of the phosphor and the fiber-optic taper to the PSF was achieved by moving the detector so that the 20 µm X-ray beam hit the very edge of the center module and examining the ‘raw’ CCD readout image. This image contained the usual ‘intact’ pixels as well as the ‘unused’ pixels on the outer edges of the CCD chip (Fig. 1 ▶, inset B). The exposure was made long enough to just overload the central pixels so that the tails of the PSF would be significant. The intact CCD pixels (which are connected to the phosphor by intact fibers) were identified using a flood-field image (Fig. 1 ▶, inset A).

**Figure 3:**
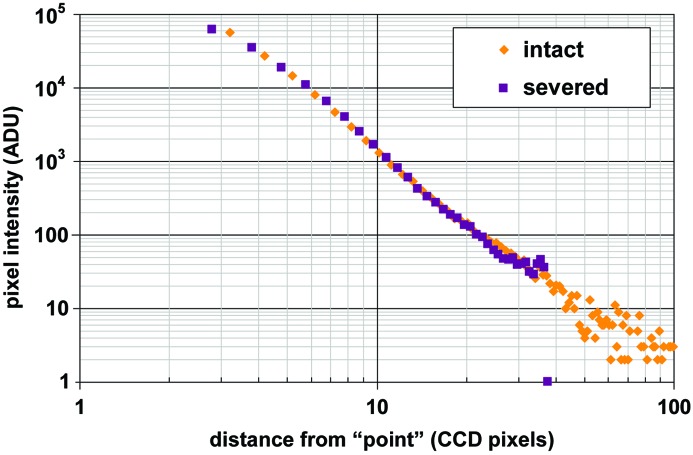
Comparison of relative intensity observed from CCD pixels attached directly to phosphor *via* intact fibers and those not connected to phosphor at all, except *via* scattering from the intact fibers. The similarity of intensity fall-off and overall scale indicates that the dominant component of the PSF tails is scattering between fibers in the taper.

The pixel values from a trace through the image shown in Fig. 1 ▶ (inset A) are plotted in Fig. 3 ▶. It should be noted that the intact and severed curves in this figure are on the same scale, but the origin of the *x*-axis was arbitrarily chosen at 0.2 pixel widths from the center of the pixel with maximum intensity on the image. The actual fractional-pixel position of the beam impact point is obviously not available from the pixel values themselves, and extrapolating it by fitting a symmetric function to the nearby tails is not appropriate here because such a fit assumes that the PSF is identical in the intact and severed regions. However, this 0.2 pixel offset brings the two traces in Fig. 3 ▶ into close coincidence, demonstrating that the rate of fall-off in intensity is identical. A similar alignment could have been achieved by applying a scale factor to one of the curves (shifting all points up or down by the same distance on this log-scale graph), but this was not done. It is possible that there is a simple scale factor difference between the intact and severed regions, but it cannot be more than the intensity change produced by moving the beam by one pixel.

### 2.3. Mathematical representation of the PSF

The pixel intensities observed here are not the ‘true’ PSF, but rather the convolution of the PSF with the beam profile, followed by integrating over the area of each pixel, so in this section we describe how these effects were decoupled. Specifically, the point spread observed here appears best described by a Moffat function (Moffat, 1969 ▶), which is essentially the convolution of a Gaussian with a power law. Unfortunately this convolution cannot be expressed in closed form, but the sum of a sufficient number of Gaussians can represent almost any function to within a desired error. A highly successful example of this approach is the popular ‘5-Gaussian’ representation of atomic scattering factors (Vand *et al.*, 1957 ▶; Cromer & Waber, 1965 ▶; Maslen *et al.*, 1999 ▶). The main utility of this representation is that convoluting atomic shapes with a Gaussian ‘blur’ may be performed analytically by simply adding the relevant *B*-factor to that of each of the component Gaussian terms.

For example, if both the PSF and the beam have Gaussian shapes, then the spot recorded on the detector will also be Gaussian, but with a full width at half-maximum (FWHM) related to that of the PSF (*w*_PSF_) and beam (*w*_beam_) by
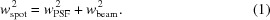
However, if either the beam or the PSF are not Gaussian, the convolution is not this simple. Suppose the PSF is still Gaussian but the X-ray beam profile is bimodal and, in effect, consists of two Gaussian ‘sub-beams’ with different *w*_beam_ and intensity. In this case the spot recorded on the detector is the sum of the two spots one would observe with either sub-beam alone, using (1) to compute the FWHM of each ‘sub-spot’. This treatment can be extended to an arbitrary number of sub-beams, and theoretically any beam shape that can be ‘painted’ onto the detector face by using a variable Gaussian beam may be modeled with this formalism. In exactly the same way the spot profile resulting from a simple Gaussian beam and a non-Gaussian PSF may be expressed as a sum of Gaussians if a suitable Gaussian-sum approximation to the PSF can be found.

Two-dimensional Gaussians may not have equal FWHMs in both directions, and indeed the major and minor axes may be tilted relative to the Cartesian coordinate system of the pixel plane. So, in general, two-dimensional Gaussians are convoluted by summing the elements of their covariance matrices.

The power-law component of the PSF found here appears to be of order 3, which resembles the solid angle subtended by a pixel at a point source of light some distance *g* above the pixel plane,
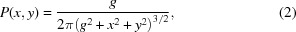
where *x* and *y* are the Cartesian coordinates of a point of interest relative to the beam impact point. The integral of *P* over the entire pixel plane is unity, reflecting how the energy of a single photon is divided amongst the pixels, and *P* may be thought of as having units of ‘intensity’ per unit area.

For comparison, the symmetric two-dimensional Gaussian with unit integral and unit FWHM is
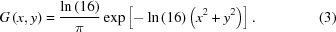
We use *G* to help represent the Gaussian component of the Moffat function,
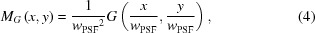
which is still centrosymmetric and normalized to integrate to unity, but has FWHM *w*_PSF_ (µm). In turn, the X-ray beam may also be taken to have a Gaussian shape, but perhaps with different FWHM in the *x* and *y* directions (*w*_beam,*x*_ and *w*_beam,*y*_),
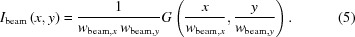
Again the integral of *I*_beam_ over the entire pixel plane is unity, since it represents the probability distribution of photon impact points.

Since both *M*_*G*_ and *I*_beam_ are Gaussians, their convolution (*M*_*G*_ ⊗ *I*_beam_) may be computed analytically using (1) (see below), but the convolution *P* ⊗ *M*_G_ cannot be expressed in closed form. We therefore approximate *P* as the sum of a number of Gaussians,
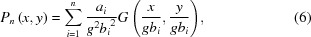
where *g* is still the height of the ‘point source’ over the pixel plane, and *a*_*i*_, *b*_*i*_ are obtained by a fit of (6) to *P* with the constraint that the sum of the volume of all *n* Gaussian terms must be equal to 1. An example of such coefficients with *n* = 8 is given in Table 1 ▶. Using these coefficients, *P*_*n*_ matches *P* to within 2.5% error over the six-decade range of the data available here.

Now, since addition and convolution commute, *P*_*n*_ ⊗ *M*_*G*_ may be expressed analytically as the sum of *n* Gaussians, and used to approximate the full PSF (*P* ⊗ *M*_*G*_). Furthermore, the full intensity spread *P* ⊗ *M*_*G*_ ⊗ *I*_beam_ may also be approximated with only *n* Gaussians. Specifically, we take each term in *P*_*n*_ individually, and substitute the squared FWHM (*g* ^2^ *b*_*i*_^2^) with the sum of the squares of all the widths involved,
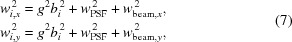
where *w*_PSF_ is the FWHM of the Gaussian component of the PSF, *w*_beam,*x*_ and *w*_beam,*y*_ are the FWHM of the beam in the *x* and *y* directions, and *w*_*i*,*x*_ is the FWHM of the *i*th Gaussian term in the approximation of *I*_point_, the intensity per unit area deposited by a photon at any point in the pixel plane,
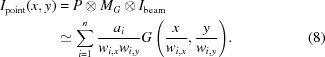
If the beam shape is more complicated than a simple Gaussian, then it too may be represented as the sum of a collection of weighted Gaussian functions, much in the same way *P* is approximated by *P*_*n*_. That is, any beam profile may be represented as the sum of a collection of *m* Gaussian-shaped sub-beams. Replacing each of these sub-beams with (8) yields a total of *nm* Gaussian terms, representing the observed spot shape on the detector. In this work, however, we restricted our representation to a simple Gaussian-shaped beam.

Now, since we find below that *g* = 27 µm, *w*_PSF_ = 76 µm and the pixel size (ℓ_pix_) is 51.27 µm, we expect that the value of *I*_point_ will vary significantly from one side of a pixel to the other. Simply evaluating *I*_point_ at the center coordinate of the pixel will not be a faithful representation of the real detector behavior, which is to integrate *I*_point_ over the whole area of the spot. Algorithmically, this may be combated by dividing each pixel into many sub-pixels and averaging, but a much more elegant and computationally expedient approach is to take advantage of the analytic expression for the integral of a Gaussian over a square,
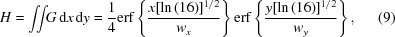
where erf is the Gauss error function, and *w*_*x*_, *w*_*y*_ are still the FWHM of ‘*G*’, the Gaussian peak being integrated. The signal expected from a pixel is computed by taking the differences between the values of *H* at the pixel corners,
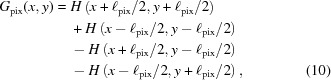
where ℓ_pix_ is the linear dimension of the edge of the square pixel (µm) and *x* and *y* are the Cartesian coordinates of the pixel center relative to the beam impact point. Note that as ℓ_pix_ becomes large relative to *w*_*x*_ and *w*_*y*_ the value of *G*_pix_(0,0) approaches unity, but as ℓ_pix_ becomes small relative to *w*_*x*_ and *w*_*y*_ the value *G*_pix_(*x*,*y*) approaches *G*(*x*,*y*)*ℓ*_pix_^2^. We may now represent the fraction of the incident beam energy deposited into a given pixel (*I*_pix_) by the convolution of a Gaussian beam shape with our Moffat function PSF integrated over a pixel centered at *x*,*y* relative to the beam center,
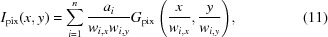
where *a*_*i*_ are taken from Table 1 ▶ and *w*_*i*,*x*_ are taken from *b*_*i*_ in Table 1 ▶ *via* (7). This function is represented by the brown lines in Fig. 2 ▶.

## 3. Results and discussion

### 3.1. X-ray beam effects

The beam shape at the detector surface was expected to be a ∼20 µm-diameter rounded top-hat profile. The uncollimated beam profile at the pinhole position is a Gaussian of ∼120 µm × 120 µm FWHM (not shown), and so is essentially flat across the 15 µm-diameter Ta pinhole. The intensity distribution passing through the pinhole must therefore be a 15 µm top-hat disk, dropping from 100% transmission to ∼1 part per billion (through 50 µm Ta) across the wall of the hole. This shape must be convoluted with the 4.2 µm square top-hat distribution arising from the 50 µrad-wide distribution of beam directions (divergence) at the detector distance of 85 mm. In addition, any irregularities in the walls of the hole must also be convoluted with the beam shape, but these are expected to be much smaller than 4 µm, so we assume here that the divergence-induced broadening dominates, giving a beam width at the baseline of ∼20 µm. This beam shape is less than half of the diameter of a pixel, but it should be noted that the PSF measured here is the convolution of the true PSF and this ∼20 µm-wide smoothed top-hat function. This deconvolution was achieved by the Gaussian decomposition described above.

The maximum possible fluorescence from the pinhole is dictated by the number of incident photons absorbed in the pinhole substrate (the Ta metal in which the hole was drilled). With a 120 µm FWHM Gaussian incident beam, 368 photons are absorbed in the pinhole substrate for every photon passing through a 15 µm-diameter hole (ratio of the numerical integral of the two-dimensional Gaussian, in and out of the central hole). If all of these 11141 eV photons are absorbed, then 30% and 60% will be taken up by the *L*_2_- and *L*_3_-edges, respectively, and ∼25% of each of these absorptions will result in a fluorescent X-ray photon emission (McMaster *et al.*, 1969–1970 ▶). Since the solid angle subtended by a 50 µm detector pixel at 85 mm from the pinhole position is 3.5 × 10^−7^ sterad, we expect one fluorescent background photon per pixel for every 4.3 × 10^5^ ‘spot’ photons. This is assuming no attenuation of the fluorescent photons (8–9 keV), which would actually be stopped 4.7 × 10^5^-fold by 50 µm of Ta. No doubt a fraction of fluorescence generated in the walls of the hole does pass through the hole, but these photons originate from a very small fraction of the total incident photons, and thus the contribution of fluorescent photons to the net signal in the ROI is negligible over our six-decade intensity range of interest.

**Figure 4:**
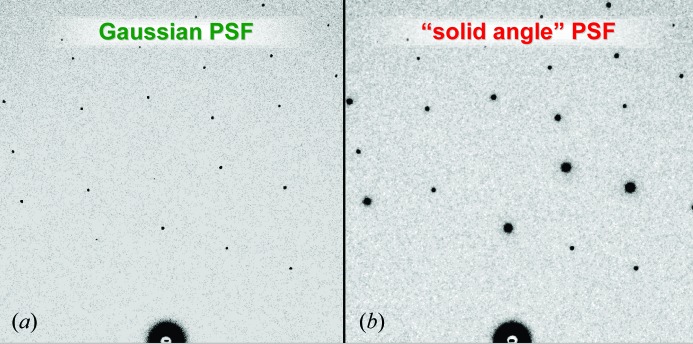
Simulated diffraction patterns from *MLFSOM* (Holton, 2008 ▶) with spots of various intensities blurred by either (*a*) a Gaussian filter or (*b*) the ‘solid angle’ PSF model proposed here. Both of these blurring filters have the same FWHM and the 16-bit images were histogram-equalized before display in *ADXV* (Arvai, 2012 ▶; Szebenyi *et al.*, 1997 ▶). The faint tails of the PSF make bright spots appear much larger than weak spots in (*b*), but not in (*a*).

Bragg scattering from the pinhole will obviously be negligible as the first diffraction order from crystalline Ta is well outside the 200 × 200 pixel region of interest used here. Small-angle X-ray scattering (SAXS) from the pinhole walls may have some contribution, but this is difficult to predict without knowing the microscopic grain structure of the laser-drilled material. However, since such small-angle contributions will also propagate through a protein crystal much like the rest of the incident beam, the overall PSF measured here is nevertheless directly relevant to protein crystallographic measurements, which are generally performed with some kind of beam-defining aperture. Indeed, applying this PSF to simulated diffraction patterns (Fig. 4 ▶) captures much of the visual appearance of spots on real images. Most of all, our observation that the PSF into ‘severed’ fibers is the same as that in ‘intact’ fibers excludes the possibility of significant contributions from pinhole fluorescence or SAXS.

No beam hardening is suspected with this set-up because the Si(333) reflection at 33423 eV is well above the high-energy cut-off of the X-ray mirrors at ∼17 keV (MacDowell *et al.*, 2004 ▶), and we have previously verified the spectral purity of the beam with an identical optical set-up (Owen *et al.*, 2009 ▶) at ALS beamline 12.3.1 [instrument described by Trame *et al.* (2004 ▶)] using an energy-resolving X-ray detector from Evex (Princeton, NJ, USA).

### 3.2. Possible origins of the inverse-cube-law PSF

The isolation of this inverse-cube dependence to the fiber-optic taper itself and the resemblance of the observed PSF to the solid angle subtended by a source 27 µm above the pixel plane suggests a scattering mechanism. This is not unexpected. Since the fibers are in physical contact with the phosphor their numerical apertures are overfilled and every possible incidence angle up to the limit of total internal reflection is represented in the captured light. The limiting rays will continue to bounce back and forth down the fiber until they encounter a bend, which will increase some of the angles with the walls beyond the total-internal-reflection angle and so some of the light escapes. Scattering is therefore an unavoidable phenomenon in fiber-optic tapers, and indeed any optical system. The almost ubiquitous application of the Moffat function in astronomy and other fields is testament to this.

The origin of the inverse-cube-law dependence may then be explained by analogy with a point light source hovering 27 µm over a plane of pixels. This system does produce an intensity distribution on the pixels given by *P* from equation (2), and continues to do so even if the space between the light source and the pixels is filled with glass beads, or other strongly scattering but low-absorbing material. *P* arises from conservation of energy, so as long as the medium is not strongly absorbing an inverse cube dependence is expected. Of course, the optical-fiber tapers are much longer than 27 µm, but the mean free path of a photon travelling through a fiber-optic taper is much longer in the direction of the fibers than it is in the directions crossing the fibers. Provided the number of ‘scattering events’ expected for an average photon moving down the taper axis from one end to the other is equal to that expected for a photon moving 27 µm normal to the fiber axes (2.7 fiber diameters), then the taper can be regarded as a ‘stretched’ pile of glass beads, and *P* still applies.

These tapers do include extra-mural absorbing fibers (EMA) to combat the effects of scattering, but cannot eliminate it entirely. It is possible that the EMA effect manifests as the slight ‘wiggle’ at about 500 µm in the blue points on Fig. 2 ▶, but we have no way to confirm this. The wiggle could also be due a slight systematic offset in the ‘zero’ reference during our averaging process, but no attempt was made here to ‘straighten’ the data by applying an arbitrary offset.

## 4. Conclusions

The PSF of the popular fiber-coupled CCD X-ray detector design is neither Gaussian nor Lorentzian, but instead is better described as a Moffat function. The observation of identical PSF into ‘severed’ as well as ‘intact’ fibers strongly suggests that the ‘tails’ of the PSF arise from the scattering of visible light in the taper only, with little influence from the phosphor and the distribution of X-rays in the spot. Since the tails are not independent X-ray events, the visual appearance of ‘bigger’ spots as they grow brighter need not be detrimental to data quality. Specifically, we expect that by fitting an expression for the spot-PSF convolution as described here directly to pixel values will result in more accurate spot intensity integrals than those currently being obtained using conventional profile-fitting methods (which assume that the intensity of a pixel is due exclusively to X-ray photons falling directly upon it). A ‘fitting approach’ would eliminate systematic errors in background estimation arising from the tails and also suppress the influence of shot noise from X-ray photons falling on pixels outside the ‘true’ spot area.

The Gaussian component of the PSF (*w*_PSF_) is estimated here to be ∼73 µm after deconvoluting a beam size of 20 µm and accounting for finite pixel size as described above. This is far too short to effectively discern a difference between ‘intact’ and ‘severed’ fibers, so the relative contribution of the taper and phosphor to this aspect of the PSF cannot be separated here. However, previous reports (Gruner *et al.*, 2002 ▶) revealed that changing phosphor thickness does have an effect on the PSF, as does more aggressive EMA strategies (such as cladding each fiber in black), so it is expected that both phosphor and fiber contribute to *w*_PSF_.

Admittedly, the measurements presented here were taken from only a single detector, but, since all commercially available fiber-coupled CCD detectors use tapers made by the same company (Incom, Charlton MA, USA) using similar EMA, it is expected that at least the functional form of the PSF presented here is generally applicable.

## Figures and Tables

**Table 1:** Coefficients used to approximate *P* with *P*_*n*_ when *n* = 8

	*i*							
	1	2	3	4	5	6	7	8
*a*_*i*_	0.1245	0.3170	0.2540	0.1474	0.07857	0.04120	0.02243	0.01494
*b*_*i*_	1.1401	2.1722	4.182	8.070	15.57	30.08	59.09	135.96
